# 1825. Comparative Effectiveness of Ampicillin versus Vancomycin or Daptomycin for the Treatment of Ampicillin-Susceptible *Enterococcus* spp. Bloodstream Infections

**DOI:** 10.1093/ofid/ofac492.1455

**Published:** 2022-12-15

**Authors:** Hubert C Chua, Kady Phe

**Affiliations:** Baylor St. Luke's Medical Center/University of Houston, Houston, Texas; Baylor St. Luke's Medical Center, Houston, Texas

## Abstract

**Background:**

Treatment options against *Enterococcus* spp., a common nosocomial pathogen, are limited due to intrinsic resistance to multiple antibiotics. Ampicillin is considered drug of choice if susceptible, but data comparing outcomes between ampicillin, daptomycin, and vancomycin for ampicillin-sensitive *Enterococcus* spp. bacteremia are limited.

**Methods:**

This was a single-center retrospective cohort study of adults with confirmed ampicillin-sensitive *Enterococcus* spp. bloodstream infection (BSI) who received definitive treatment with intravenous ampicillin, daptomycin, or vancomycin from January 2013 to December 2021. Polymicrobial BSI were excluded. Definitive treatment was defined as the active antibiotic used for more than 50% of the total treatment duration following availability of susceptibility results. The primary outcome was 28-day all-cause hospital mortality; secondary outcomes were 90-day readmission due to microbiological recurrence and all-cause hospital mortality. Multivariate Cox regression analysis was performed to identify independent risk factors for 28-day hospital mortality.

**Results:**

A total of 199 patients (ampicillin, n = 141; daptomycin, n = 17; vancomycin, n = 41) were evaluated. Overall in-hospital all-cause mortality was 15.1% (n = 30). Twenty-eight-day hospital mortality was numerically lower with ampicillin and daptomycin compared to vancomycin (9.2%, 11.8%, and 22%, respectively; p = 0.088). Ninety-day readmission due to microbiological recurrence (4.3%, 5.9%, and 7.3%; p = 0.722) and all-cause hospital mortality (12.8%, 17.6%, and 22%; p = 0.335) were not significantly different between treatment groups. Independent risk factors for 28-day hospital mortality included age (hazard ratio [HR] 1.05, 95% confidence interval [CI] 1.02 to 1.09; p = 0.003), *E. faecium* BSI (HR 3.94, 95% CI 1.39 to 11.1; p = 0.010), and definitive treatment with vancomycin (HR 2.54, 95% CI 1.04 to 6.21; p = 0.040).

**Conclusion:**

Vancomycin used as definitive therapy for ampicillin-sensitive *Enterococcus* spp. bacteremia was found to be an independent risk factor for 28-day mortality. Further studies evaluating the role of daptomycin in this setting are warranted.

**Disclosures:**

**All Authors**: No reported disclosures.

